# Characterization of Atg38 and NRBF2, a fifth subunit of the autophagic Vps34/PIK3C3 complex

**DOI:** 10.1080/15548627.2016.1226736

**Published:** 2016-09-14

**Authors:** Yohei Ohashi, Nicolas Soler, Miguel García Ortegón, Lufei Zhang, Marie L. Kirsten, Olga Perisic, Glenn R. Masson, John E. Burke, Arjen J. Jakobi, Apostolos A. Apostolakis, Christopher M. Johnson, Maki Ohashi, Nicholas T. Ktistakis, Carsten Sachse, Roger L. Williams

**Affiliations:** a MRC Laboratory of Molecular Biology, Cambridge, United Kingdom; b European Molecular Biology Laboratory, Structural and Computational Biology Unit, Heidelberg, Germany; c European Molecular Biology Laboratory, Hamburg Unit, Hamburg, Germany; d The Babraham Institute, Cambridge, UK

**Keywords:** Atg14, Atg38, Beclin 1, complex I, crystal structure, EM structure, NRBF2, Vps15, Vps30, Vps34

## Abstract

The phosphatidylinositol 3-kinase Vps34 is part of several protein complexes. The structural organization of heterotetrameric complexes is starting to emerge, but little is known about organization of additional accessory subunits that interact with these assemblies. Combining hydrogen-deuterium exchange mass spectrometry (HDX-MS), X-ray crystallography and electron microscopy (EM), we have characterized Atg38 and its human ortholog NRBF2, accessory components of complex I consisting of Vps15-Vps34-Vps30/Atg6-Atg14 (yeast) and PIK3R4/VPS15-PIK3C3/VPS34-BECN1/Beclin 1-ATG14 (human). HDX-MS shows that Atg38 binds the Vps30-Atg14 subcomplex of complex I, using mainly its N-terminal MIT domain and bridges the coiled-coil I regions of Atg14 and Vps30 in the base of complex I. The Atg38 C-terminal domain is important for localization to the phagophore assembly site (PAS) and homodimerization. Our 2.2 Å resolution crystal structure of the Atg38 C-terminal homodimerization domain shows 2 segments of α-helices assembling into a mushroom-like asymmetric homodimer with a 4-helix cap and a parallel coiled-coil stalk. One Atg38 homodimer engages a single complex I. This is in sharp contrast to human NRBF2, which also forms a homodimer, but this homodimer can bridge 2 complex I assemblies.

## Introduction

Vps34, the primordial phosphatidylinositol 3-kinase (PtdIns3K) that phosphorylates the 3-OH of phosphatidylinositol to form phosphatidylinositol 3-phosphate (PtdIns3P), is conserved in all eukaryotes, and it is important both for endocytic traffic to lysosomes and vacuoles and for autophagy.^1-3^ PtdIns3P, which is targeted by PtdIns3P-binding proteins such as Atg18 (WIPI1/2/3/4 in mammalian cells), is essential for autophagosome creation.^4,5^

In cells, it is unlikely that Vps34 ever works alone. The minimal Vps34 complex is a heterodimer of Vps34 with Vps15, a putative protein kinase. This Vps34-Vps15 complex is the core for 2 larger, heterotetrameric assemblies known as complexes I and II. Complex I is involved in autophagy, whereas complex II is involved in endocytic pathways. In addition to the Vps34-Vps15 core, complex I includes an accessory pair of proteins, Vps30/Atg6 (BECN1 in mammals) and Atg14 (ATG14 in mammals).^6^ In contrast, complex II contains 3 of the same subunits (Vps34, Vps15 and Vps30), but Vps38 (UVRAG in mammals) replaces Atg14. The structure of complex II shows that Vps30 and Vps38 form a parallel coiled-coil heterodimer that clamps onto the Vps15-Vps34 core in such a way that both Vps30 and Vps38 interact with Vps15.^7^

A fifth subunit of complex I, Atg38, has been described in yeast, and in vivo experiments suggested that this recently discovered subunit is important for the integrity of complex I.^8^ Araki et al. postulate that Atg38 is orthologous to NRBF2 in mammals,^8^ and 3 recent studies in mammalian cells support this proposition.^9-11^ All studies agree that Atg38/NRBF2 binds to complex I, and that this subunit localizes to phagophores during starvation. However, there are several discrepancies among the reported results. Lu et al. show that NRBF2 binds to both complex I and complex II,^10^ whereas others have shown that Atg38/NRBF2 is complex I-specific.^8,9,11^ It has been reported that NRBF2 is critical for induction of autophagy,^9^ while one report indicates that NRBF2 suppresses autophagy.^11^ Also, each study identifies different components of complex I as binding partners of Atg38/NRBF2. Araki et al. show that Atg38 binds to Atg14 and Vps34,^8^ while another study reports NRBF2 binds to the WD40 domain of VPS15, but not to either BECN1 or ATG14,^9^ Other reports show NRBF2 binds to ATG14.^10,11^ These ambiguities might have been caused by employing different experimental systems and using immunoprecipitates rather than purified proteins.

The domain organization of Atg38 and NRBF2 sheds some light on how these proteins might function. Both Atg38 and NRBF2 have an N-terminal microtubule-interacting and targeting (MIT) domain and a C-terminal region of unknown structure.^8^ The MIT domain of Atg38 and NRBF2 is important for binding to complex I and for functionality during starvation.^8,10,11^ Compared to the MIT domain, the function of the C-terminal region remains unclear except that it has a role in homodimerization.^8,10,11^

In order to clarify how Atg38 and NRBF2 interact with complex I and to understand the consequences of these interactions, we compared the structural organization of complex I in the presence and absence of Atg38/NRBF2. We find that stable yeast and human complex I can be purified both in the presence and absence of Atg38/NRBF2. Using hydrogen-deuterium exchange mass-spectrometry (HDX-MS), we mapped interactions between the MIT domain of Atg38 and the coiled-coil I regions of Atg14 and Vps30 in the Atg14-Vps30 arm of complex I. The second arm of complexes I and II, consisting of the common Vps15-Vps34 core, does not appear to directly interact with Atg38 in complex I. Moreover, we determined the 2.2 Å resolution crystal structure of the C-terminal region of Atg38, revealing a homodimeric α-helical structure with a 4-helix bundle on top of an asymmetric coiled-coil stalk. In vivo experiments suggest that this C-terminal region is important for homodimerization, binding to complex I, localization to the PAS and potential membrane interaction. We also show that the yeast Atg38 and human NRBF2 homodimers have striking differences in their abilities to dimerize complex I.

## Results

### Yeast Vps15, Vps34, Vps30 and Atg14 form a 1:1:1:1 heterotetramer that interacts with Atg38

**Figure 1.**
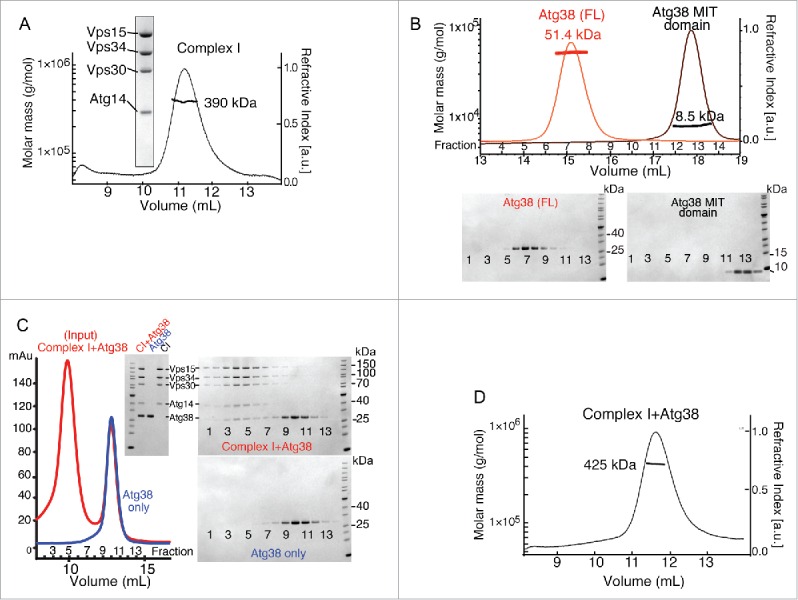
Stable heterotetrameric and heteropentameric assemblies of yeast complex I. (A) SEC-MALS analysis of purified recombinant heterotetrameric *S. cerevisiae* complex I on an S200 10/30 gel filtration column. The inset shows SDS-PAGE of the purified complex stained with InstantBlue. (B) SEC-MALS analysis of full-length Atg38 (red trace) and the Atg38 MIT domain (residues 1 to 78) (black trace) on a S75 10/30 column. The mass of full-length Atg38 is consistent with a dimer. The mass of the MIT domain alone is consistent with a monomer (calculated mass 9 kDa). (C) Purified heterotetrameric complex I was mixed with an excess of purified Atg38 and analyzed by gel filtration on a S200 10/30 column (red trace). For comparison, purified Atg38 alone was run on the same column (blue trace). The fractions were run on a SDS-PAGE and the gel was stained with InstantBlue (right). Note that the elution volumes for (A) and (C) differ since 2 different S200 columns were used. Inset: starting material of complex I+Atg38 (CI+Atg38). Also, complex I alone (CI) and Atg38 alone before mixing are loaded as indicated. (D) SEC-MALS analysis of complex I + Atg38 on a S200 10/30 column. a.u., arbitrary units; mAu, milli Absorbance units.

To gain insight into the organization and function of Vps34 complexes, we coexpressed the core 4 subunits of *S. cerevisiae* complex I in yeast. The purified complex shows stoichiometric ratios of the 4 components by SDS-PAGE analysis. Size exclusion chromatography coupled with multi-angle light scattering (SEC-MALS) shows a monodisperse peak with molecular mass of 390 kDa consistent with a 1:1:1:1 complex (expected mass is 379 kDa and calibration of the SEC-MALS with known standards indicates an error of less than 5%) (Fig. 1A).

To examine the interaction of Atg38 with complex I, we first purified Atg38 on its own. SEC-MALS analysis of purified Atg38 reveals a monodisperse peak with a molecular mass of 51.4 kDa (Fig. 1B). Given a monomeric mass of 26 kDa for Atg38, this suggests that the protein forms a homodimer. We then mixed purified heterotetrameric complex I with a 6-fold excess of purified Atg38 and analyzed the mixture by gel filtration. Atg38 was clearly incorporated into the complex I peak as shown by SDS PAGE analysis of the gel filtration fractions and was cleanly separated from the free Atg38 peak (Fig. 1C). The gel-filtration purified heteropentameric complex I was then analyzed by SEC-MALS. This analysis shows a monodisperse peak with a molecular mass of 425 kDa (Fig. 1D), suggesting that one homodimer of Atg38 binds to one heterotetrameric complex I (expected mass of 431 kDa).

Given that an Atg38 homodimer can interact with one complex I, we asked whether this stoichiometry was simply a consequence of reconstitution with an excess of Atg38 or whether this stoichiometry is maintained independently of the reconstitution conditions. Regardless of whether we use 6-fold molar excess complex I or 6-fold excess of Atg38, our gel filtration analysis shows only one peak containing both complex I and Atg38 (Fig. S1). SDS PAGE analysis of the gel filtration peak of the heteropentamer formed by mixing complex I with a 6-fold molar excess of Atg38 indicates a stoichiometry of 1:1:1:1:2 (Fig. 1C and S1), consistent with the SEC-MALS analysis shown in Fig. 1D. When the heteropentamer is reconstituted in a condition with a 6-fold molar excess of complex I compared with Atg38, the SDS PAGE is consistent with a mixture of predominately heterotetrameric complex I and a minor population of heteropentamer (Fig. S1). Since there is no evidence of a larger complex induced by Atg38, these results indicate that Atg38 homodimers preferentially interact with only one complex I and do not link 2 heterotetramers.

### Interaction of human complex I with NRBF2

**Figure 2.**
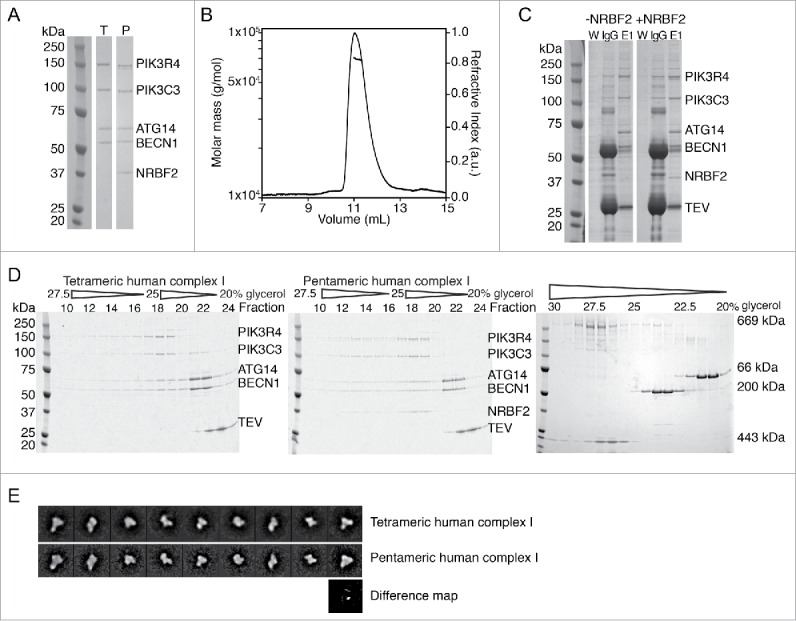
Stable heterotetrameric and heteropentameric assemblies of human complex I. (A) SDS gels of purified human heterotetrameric (T) and heteropentameric (P) complexes coexpressed in HEK293T cells. (B) SEC-MALS profile of recombinantly expressed NRBF2, confirming it forms a dimer of 67 kDa. (C) IgG affinity isolation of complexes. The heteropentameric complex was reconstituted in vitro starting with a HEK293T lysate expressing the heterotetrameric complex (left) to which excess NRBF2 was added (right) (W: wash, IgG: beads, E1: elution fraction 1 after TEV cleavage from the IgG resin). (D) Glycerol gradient profiles of human complexes (left and middle gels). Both have comparable migration profiles, indicating a similar size. Right gel: glycerol gradient profiles of standard markers. 669 kDa: Thyroglobulin; 443 kDa: Apoferritin; 200 kDa: β-Amylase; 66 kDa: Bovine serum albumin. (E) Comparison of electron microscopy class averages of the heterotetrameric (top row) and heteropentameric complex (bottom row) embedded in negative stain show that the overall V-shaped architecture of both complexes is very similar. In particular views, there is additional density of NRBF2 detectable at the base of the complex, as confirmed by the difference map below. a.u., arbitrary units.

In order to better understand the role of the human ortholog of Atg38, NRBF2, in the context of the human complex I, we transiently expressed and purified the corresponding heterotetrameric complex in HEK293T cells (Fig. 2). SDS PAGE reveals that the purified complex contains all expected components at stoichiometric ratios (Fig. 2A, lane T). SEC-MALS indicates that free NRBF2 has a mass of 67 KDa (Fig. 2B), confirming that the 32.5 kDa NRBF2 forms a homodimer, analogous to Atg38. We tested the incorporation of NRBF2 into the heterotetrameric human complex I both by expressing all 5 components together in HEK293T cells or by adding purified NRBF2 to HEK293T lysates expressing the heterotetrameric complex. SDS PAGE shows that NRBF2 incorporates efficiently into the human heterotetrameric complex either when coexpressed (Fig. 2A) or when reconstituted (Fig. 2C). Glycerol gradient ultracentrifugation of both the heterotetrameric and heteropentameric complexes showed that most of the material migrates in the same density of approximately 25% glycerol (Fig. 2D).

We asked whether the size of the human heteropentamer is influenced by the relative abundance of NRBF2. When a 6-fold molar excess of complex I is mixed with NRBF2, SEC-MALS shows only 2 peaks (Fig. S2, blue trace). The first peak (fractions 2 and 3), with larger mass than complex I alone, has an average mass most consistent with 2 complexes linked by an NRBF2 dimer, and the second peak (in fractions 7 and 8) appears to consist of complex I alone. In contrast, when a 6-fold molar excess of NRBF2 is mixed with human complex I and analyzed by SEC-MALS, 2 new higher mass peaks appear compared with individual components alone (red trace Fig. S2). The first peak (in fractions 2 and 3, Fig. S2) with the largest mass is most consistent with 2 heterotetrameric complexes linked by an NRBF2 homodimer. The second peak (fraction 5, Fig. S2) shows a mass of 481 kDa, which is closest to the expected mass of one complex I with one NRBF2 homodimer (calculated mass 437 kDa). The fourth peak consisting of NRBF2 dimer alone (Fraction 11) is well separated from the heteromeric complexes. These results suggest that in contrast to yeast Atg38, one NRBF2 homodimer can bridge 2 copies of human complex I. Although the MIT domain of NRBF2 interacts with complex I, the MIT domain on its own cannot bridge 2 complexes. When human complex I was mixed with NRBF2 MIT domain (ratio of 6 complex I:1 MIT) and analyzed by gel filtration (Fig. S3), the complex I-MIT mixture eluted as a single peak at nearly the same volume as complex I alone. SDS PAGE analysis of the peak shows all 5 components. This shows that the monomeric MIT domain cannot link 2 complex I assemblies.

Using negative staining EM analysis of GraFix^12^ cross-linked heterotetrameric and heteropentameric human complexes (Fig. 2E), we examined the homogeneous fractions. This material consists of predominantly single asymmetric assemblies (Fig. S4) with an overall V-shape as reported previously.^13^ Additional density visible at the base of the V in some class averages suggests interaction of NRBF2 with the heterotetrameric complex (Fig. 2E).

### Mapping the Atg38-complex I interface by HDX-MS

**Figure 3.**
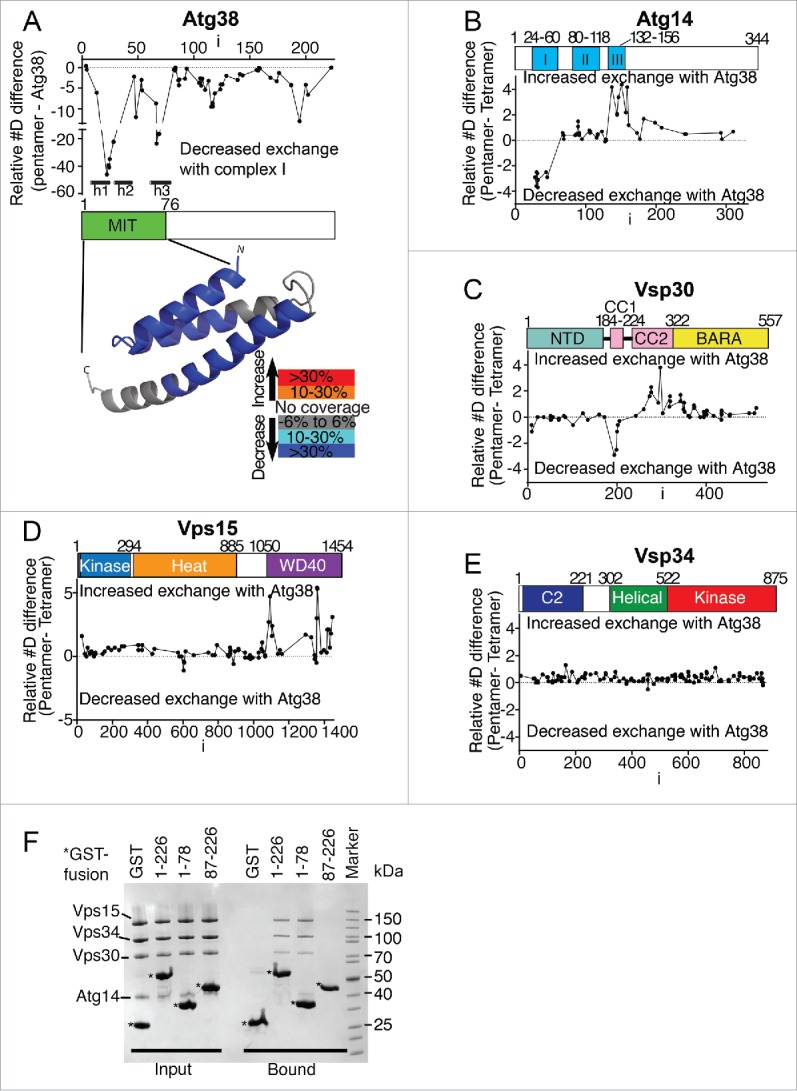
Mapping the Atg38-complex I interface by HDX-MS. (A to E) Changes in HDX between heterotetrameric complex I, complex I+Atg38 and free Atg38. (A) Differences in HDX for Atg38 between free Atg38 and Atg38 in the heteropentameric complex I. A model for the N-terminal MIT domain is illustrated below with HDX differences mapped onto it. (B to E) Differences in HDX between heterotetrameric complex I and heteropentameric complex I for Atg14 (B), for Vps30 (C), for Vps15 (D) and for Vps34 (E). (F) GST affinity isolation experiments with various deletion variants of Atg38.

We mapped the binding between the yeast complex I and Atg38 using hydrogen-deuterium exchange mass-spectrometry (HDX-MS), which provides information related to solvent accessibility for protein backbone amide hydrogens. This technique enables mapping of protein-protein interactions in solution without requiring any labeling, mutagenesis or truncation variants.^14,15^ We determined HDX for 3 entities: the heterotetrameric complex I (Vps15-Vps34-Vps30-Atg14), free Atg38 and complex I with Atg38 (the heteropentamer was reconstituted in the presence of excess Atg38 and gel filtered; the peak with complex I bound to Atg38 was concentrated and used for HDX-MS). Differences in HDX among these states provide information related to their relative accessibilities (Fig. 3). In Atg38, the 3 predicted helices of the N-terminal MIT-like domain were highly protected from exchange in the presence of complex I, suggesting that this region participates in binding to complex I (Fig. 3A). This is consistent with analysis of Atg38 deletion variants showing that the minimal construct that binds complex I is the MIT domain alone (residues 1 to 78, Fig. 3F).

Two regions in the C-terminal domain of Atg38 are also more protected from exchange in the presence of complex I (Fig. 3A). Although this domain could directly bind complex I, GST affinity isolation assays indicate that the C-terminal region alone (residues 87 to 226) is not sufficient for in vitro binding (Fig. 3F). Alternatively, since the C-terminal domain of Atg38 is important for forming a homodimer, the dimeric Atg38 binding to complex I could rigidify the C-terminal region, leading to decreased exchange. It may be that dimeric Atg38 increases avidity of the interaction for complex I, and that the C-terminal region is important for this dimeric avidity, but this role for the C-terminal domain would be masked in the GST affinity isolation analysis, since GST itself can form dimers.^16^

Atg14 contains 3 coiled-coil motifs (coil I, II, and III).^17^ We found that coiled-coil I (residues 25 to 36) was highly protected in the presence of Atg38 (Fig. 3B), suggesting that this region of Atg14 interacts with Atg38. We also found that the coiled-coil I of Vps30 (residues 188 to 210^7^) was protected in the presence of Atg38 (Fig. 3C). Because it is known that coiled-coil II of Atg14 binds the coiled-coil of Vps30,^17^ this change in HDX for Vps30 could be due either to a direct interaction with Atg38 or it may reflect Atg38 reinforcing the interaction between Vps30 and Atg14. Besides protections, several regions become more exposed in the presence of Atg38: the coiled-coil III of Atg14 (Fig. 3B), the last half of coiled-coil II of Vps30 (Fig. 3C) and several regions in the WD40 domain of Vps15 (Fig. 3D; residues 1076 to 1112, 1352 to 1363, 1406 to 1429, and 1442 to 1449). Based on the crystal structure of yeast complex II^7^ and the 28 Å resolution negative-stain EM structure of human complex I,^13^ these regions map to a single area in complex I, which may undergo some conformational rearrangements within complex I upon Agt38 binding. Although it was reported that Atg38 binds Vps34 based on yeast 2-hybrid analysis,^8^ we found no significant differences in HDX in Vps34 (Fig. 3E). The Vps34 interaction detected in the 2-hybrid analysis might not have been a direct interaction. However, we do not exclude the possibility that the regions of Vps34-Vps15 for which we have no coverage in the HDX-MS analysis (10% of the residues in Vps34 and 40% in Vps15) may contribute to interaction with Atg38. We determined a 28-Å resolution structure of the isolated Vps15-Vps34 heterodimer (Fig. S5), which suggests that the structure of this heterodimeric core is similar to what is observed in complex II that does not bind Atg38.

### Crystal structure of the C-terminal homodimerization region of Atg38

To gain insight into the role of the C-terminal region of Atg38, we determined its structure by X-ray crystallography. We obtained crystals using full-length Atg38 *S. cerevisiae* protein that had been subjected to limited proteolysis with subtilisin. The structure was solved by multiwavelength anomalous dispersion (MAD) using selenomethionine-derivatized protein and was refined to 2.2 Å resolution (Table 1). The structure showed that the crystallized region corresponds to residues 119 to 211 in the Atg38 C-terminal domain.

**Table 1.** Data collection, phasing and refinement statistics*.

Data collection	Native (MGOx11)	SeMet (MGOx65)	
Space group	P2_1_2_1_2	P2_1_2_1_2	
Cell dimensions			
*a, b, c* (Å)	81.38, 249.28, 50.20	81.27, 249.95, 50.28	
α, β, γ (°)	90, 90, 90	90, 90, 90	
		*Peak*	*Inflection*
Wavelength	0.976260	0.979030	0.979320
Resolution (Å)	2.2 (2.27–2.2)	2.5 (2.6–2.5)	2.5 (2.6–2.5)
*R*_sym_ or *R*_merge_	0.062 (0.891)	0.11 (1.39)	0.077 (0.82)
I / σI	16.9 (2.3)	16.1 (2.2)	21.9 (3.4)
Completeness (%)	99.8 (99.6)	99.7 (99.7)	99.8 (99.8)
Redundancy	6.6 (6.3)	13.0 (13.8)	13.1 (13.8)
**Refinement**			
Resolution (Å)	2.2		
No. reflections	52821		
*R*_work_ / *R*_free_	0.20 / 0.24		
No. atoms			
Protein	4380		
Ligand/ion	144		
Water	43		
*B*-factors (Å^2^)			
Protein	84.03		
Ligand/ion	79.37		
Water	58.38		
R.m.s deviations			
Bond lengths (Å)	0.0054		
Bond angles (°)	0.769		

* Numbers in parentheses represent statistics for the highest resolution shell.

**Figure 4.**
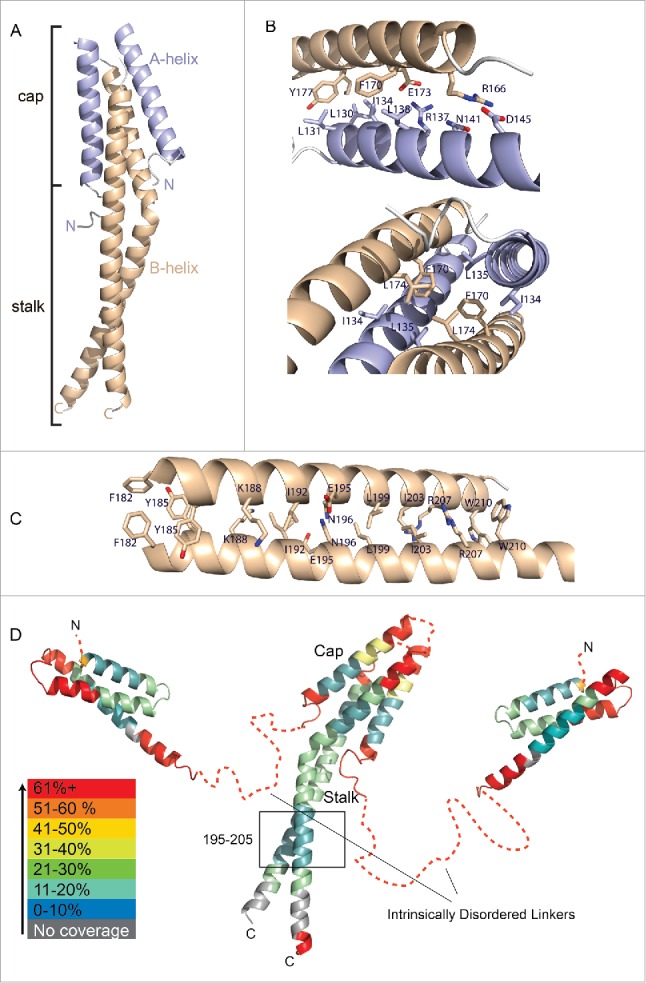
Crystal structure of the C-terminal domain of Atg38. (A) Overall structure of the homodimer. (B) Close-up view showing the interactions of helix A with the straight helix B in the cap region (upper panel) and the asymmetric A-B contacts in the cap (lower panel). (C) Close-up view of the intersubunit interactions in the stalk. (D) Helix-helix interactions between subunits result in decreased global HDX-MS, while the linker connecting the MIT domain to the C-terminal domain shows rapid exchange consistent with intrinsic disorder.

The asymmetric unit has 3 copies of an Atg38 homodimer (Fig. 4A). The 3 homodimers in the asymmetric unit are related by a 3-fold non-crystallographic rotational axis that is approximately parallel to the *b* axis of the unit cell. Because each of the 3 copies of the homodimer are very similar, and because the molecule is a homodimer in solution, our description will refer to a single homodimer. The ordered parts of the monomers are entirely helical, with a short N-terminal helix A (residues 129 to 150) folded back over a part of a longer helix B (residues 164 to 211). Each homodimer is strikingly asymmetric with the long helix B in one monomer almost straight, while helix B in the other monomer is bent at residue F182 at an angle of approximately 40°. The turn between helices A and B is not visible in the electron density. Due to the lack of continuity in the electron density between the short helix A and the long helix B of each monomer, the connection between helix A and helix B is ambiguous.

The parallel arrangement of the 2 molecules in a dimer gives rise to a 4-helix bundle at the N-terminal end of the dimer and a long, parallel coiled-coil at the C-terminal end, imparting on the dimer the overall look of a mushroom. We will refer to the segment of the homodimer corresponding to the 4-helix bundle as the cap and the protruding coiled-coil as the stalk (Fig. 4A) and the disordered residues 212 to 226 as the C-terminal tail. Overall, there are 2 homodimerization interfaces in the C-terminal region, one in the cap segment (Fig. 4B) and the other in the stalk segment (Fig. 4C), both of which we found are important for efficient homodimerization *in vivo* (see below).

Contacts in the cap include clusters of hydrophobic residues and 2 salt bridges. The asymmetry of the dimer is manifest in the contacts within the cap. In the cap, residues L130 to D145 of helix A contact residues R166 to K180 in helix B (Fig. 4B, upper panel). These contacts include 2 salt links: R166 with D145 and R137 with E173. However, because of the asymmetry of the homodimer, these salt links only involve E173 and R166 of the straight helix B and not the kinked helix B. The asymmetry of the cap is also apparent in the hydrophobic contacts. For example, at the center of the cap, residues F170 and L174 in the straight helix B contact the corresponding residues of the kinked helix B (Fig. 4B, lower panel). F170 also contacts L135 in helix A, However, the asymmetry of the cap means that other aspects of the environment of L135 are different for the 2 molecules. This asymmetry of the homodimerization disappears in the stalk (Fig. 4C).

To estimate the overall flexibility of Atg38, we used HDX-MS to measure the global exchange of the isolated Atg38 subunit. In order to map the HDX-MS results on the structure, we combined a homology model for the Atg38 N-terminal MIT domain based on the N-terminal MIT domain of NRBF2 (PDB entry 2CRB), with the crystal structure of the C-terminal domain (Fig. 4D). The HDX heat map clearly shows that the linker between these domains is disordered (warm colors, Fig. 4D). Conversely, the middle region of the MIT domain, the bottom half of the cap segment, and the residues 195 to 205 of the stalk were protected. HDX-MS shows that as with the N-terminal MIT domain, the C-terminal domain of Atg38 becomes protected upon incorporation into complex I (Fig. 3A), probably due to stabilization of the subunit and possibly due to direct interactions with complex I.

### *In vivo* characterization of the C-terminal region of Atg38

**Figure 5.**
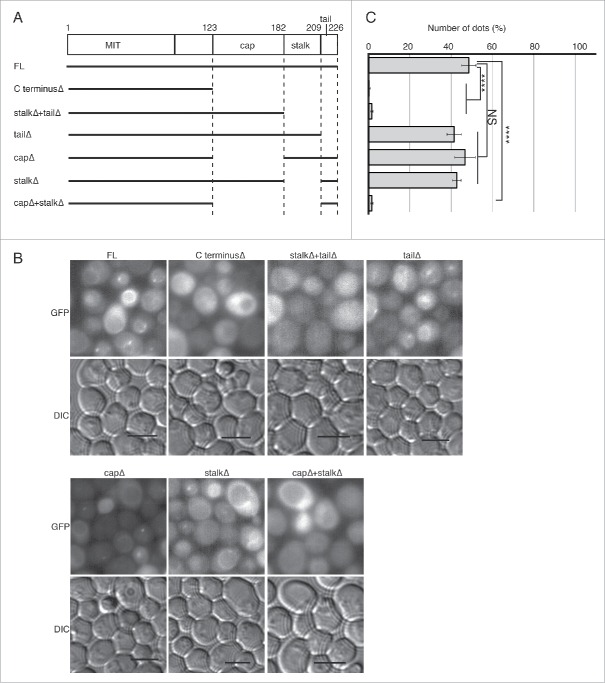
Effect of Atg38 C-terminal truncations on cellular localization. (A) Schematic representation of C-terminal truncations of Atg38. Genomic DNA fragments containing the *ATG38* promoter region (-997 base pairs) and a deletion series of *ATG38* were C-terminally fused to an EGFPx2 coding fragment on a CEN plasmid (pRS416). (B) Micrographs of GFP and differential interference contrast (DIC) channels of the Atg38 truncations. The plasmids carrying the truncations described in (A) were transformed into *atg38*Δ cells. Cells were grown to mid-log phase (OD_600_ = 0.8–1.0), then shifted to SD(-N) for 4 h, then subjected to microscopy. Scale bars: 5 µm. (C) Numbers of dots (%) were counted from triplicates of 100 cells/construct ± SD. The graph is a representative of 2 independent experiments. ****, P < 0.0001 compared to full length (FL); NS, Not Significant (P > 0.05) compared to FL.

We attempted to characterize the role of the C-terminal region of Atg38 in vivo. Genomic DNA fragments carrying deletions in the C-terminal region were C-terminally fused to a 2xEGFP tag to visualize their localization (Fig. 5A). As reported previously, the full-length Atg38 showed PAS localization (dots) during nitrogen starvation^8^ (Fig. 5B). We found that deletions of the cap (123 to 181Δ), the stalk (182 to 209Δ) or the C-terminal tail (209 to 226Δ) showed a similar number of dots as the full-length protein during nitrogen starvation (Fig. 5B, C). On the other hand, when more than one segment was deleted simultaneously (C terminusΔ, stalkΔ+tailΔ, and capΔ+stalkΔ), almost no dots were observed (Fig. 5B,C). These results suggest that the Atg38 C-terminal region is important for the localization to the PAS, and that the 3 segments in the C-terminal region may work synergistically to localize to the PAS.

**Figure 6.**
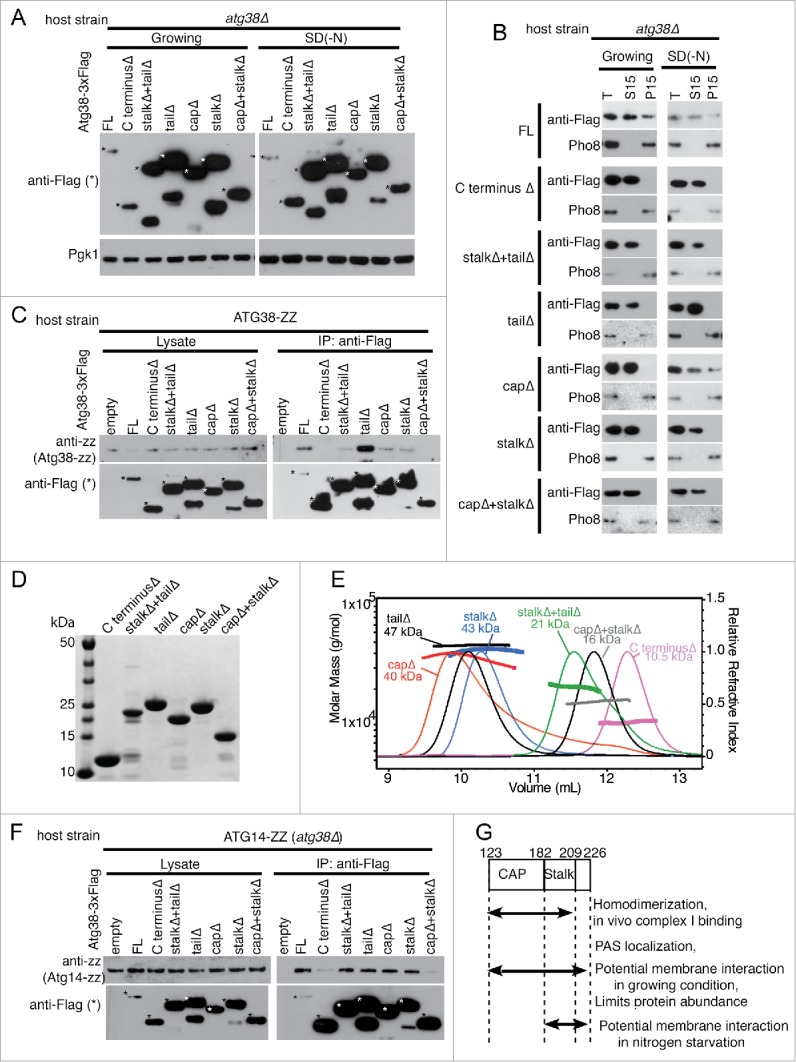
Roles of the Atg38 C-terminal domain in dimerization, interaction with complex I and cellular fractionation. (A) All Atg38 C-terminal truncations cause overexpression of the Atg38 constructs. Total cell lysates from growing and nitrogen starvation conditions were subjected to western blotting. The same C-terminal deletion fragments as used in Fig. 5 were C-terminally tagged with 3X FLAG on a CEN plasmid (pRS416). The plasmids were transformed into *atg38*Δ cells, and grown to mid-log phase (OD_600_ = 0.8–1.0; Growing), then shifted to SD(-N) for 4 h. Total cell lysates were prepared as described in Materials and Methods. Asterisks indicate proteins of interest. Pgk1 expression was used as a loading control. (B) Atg38 C-terminal truncations cause dissociation of the proteins from the P15 pellet. Subcellular fractionation of the same proteins as in (A). Cells were grown to mid-log phase (OD_600_ = 0.8–1.0; Growing), then shifted to SD(-N) medium and the total cell lysates (T), and supernatant (S15) and pellet (P15) fractions were analyzed. Pho8 was used as a membrane fraction (P15) control. (C) The C-terminal, crystallized region is important for homodimerization of Atg38. Immunoprecipitation between Atg38-ZZ and the same Atg38 truncations used above during nitrogen starvation. Yeast strain with chromosomally ZZ-tagged ATG38 was transformed with the same plasmids as (A) and (B). Cells were grown to mid-log phase, then shifted to SD(-N) for 4 h. The truncated proteins were affinity isolated with anti-Flag antibody (IP) then, the interacting Atg38-ZZ was detected with anti-ZZ antibody). The Atg38 (Full length, FL, and deletion series) proteins were detected with anti-Flag antibody. (D) SDS PAGE analysis of Atg38 deletion variants expressed in *E. coli.* (E) SEC-MALS analysis of the deletion variants shown in (D) carried out on a Superdex 75 10/30 gel filtration column. Variants with a single segment deleted have masses that are consistent with dimers (capΔ, stalkΔ and tailΔ). Constructs with 2 or 3 segments deleted have monomeric masses (stalkΔ+tailΔ, capΔ+stalkΔ and C terminusΔ). The C terminusΔ construct consists of residues 123 to 226, corresponding to a calculated mass of 14 kDa. Both SEC-MALS and intact mass spectrometry (Fig. S17) are consistent with a mass of approximately 11 kDa, suggesting that there has been some proteolysis of the construct during expression or purification from *E. coli.* (F) The crystallized region is important for the binding to Atg14. Immunoprecipitation between Atg14-ZZ and the Atg38 deletion series during nitrogen starvation. The *atg38*Δ strain, in which the ATG14 gene was chromosomally integrated with ZZ-tag at its C terminus, was transformed with the same plasmids as above. Cells were grown to mid-log phase, then shifted to SD(-N) for 4 h. The Flag-tagged truncations were affinity isolated with anti-Flag antibody (IP) and the interacting ZZ-tagged protein in the immunoprecipitates was detected with an anti-ZZ antibody, while Atg38 was detected with anti-Flag antibody. (G) Summary of in vivo experiments with Atg38 truncation variants.

To characterize the function of the C-terminal region in cells, the same deletion fragments as illustrated in Fig. 5A were fused to a 3xFLAG tag on a CEN plasmid (pRS416) and transformed into the *atg38*Δ mutant. We found that all deletions were expressed at higher levels in growing and nitrogen starvation conditions (Fig. 6A). Subcellular fractionation experiments in growing condition showed that the full-length Atg38 was detected in supernatant (S15) and pellet (P15) fractions, whereas all of the deletion proteins were predominantly detected in S15 (Fig. 6B). This suggests that each segment of the C-terminal region is important for association with membranes or larger protein complexes. In nitrogen starvation, we observed similar fractionation patterns except that the capΔ mutant was also present in the P15 fraction, suggesting that the stalk and tail segments are important for association with membranes or larger protein complexes during starvation. In the structure of the Atg38 C-terminal domain homodimer, both the cap and stalk are involved in homodimerization (Fig. 4).

Immunoprecipitation of the C-terminal deletion constructs when expressed in yeast with full-length double protein A-tagged Atg38 (Atg38-ZZ) showed that deletion of the entire C-terminal region (123 to 226Δ) or deletion of the cap and stalk together eliminates dimerization, while deletion of a single element (cap, stalk or tail) still enables some dimer formation (Fig. 6C). This is confirmed by expression of recombinant proteins in *E. coli* and SEC-MALS of the constructs, which shows that constructs with a single element deleted (capΔ, stalkΔ or tailΔ) remain dimeric, while constructs with 2 or 3 elements deleted are monomeric (Fig. 6D,E). Both the cap and stalk segments are also important for binding to complex I (Atg14-ZZ), as we saw the interactions were decreased for either the C terminusΔ or the capΔ+stalkΔ mutants (Fig. 6F). In summary, the Atg38 C-terminal region is important for PAS localization, homo-dimerization and binding to complex I, as summarized in Fig. 6G.

## Discussion

### The lipid kinase Vps34 can form 2 stable complex I assemblies

We show that 2 distinct, homogeneous, monodisperse yeast complex I assemblies can be purified. One is a heterotetrameric complex I containing equimolar ratio of the 4 subunits, Vps15-Vps34-Vps30-Atg14. The other is a heteropentameric complex I, with a dimer of the Atg38 subunit binding to the heterotetrameric complex I. Since recombinant complex I can be purified in the absence of Atg38, it does not seem that Atg38 is essential for assembly of the complex. This is further supported by the relatively mild autophagy defect elicited by the *atg38*Δ mutation compared with mutants lacking the other subunits of the complex I.^8^ Our results showing that mammalian complex I can be purified either with or without NRBF2 is also consistent with the weak phenotype of the *nrbf2*^−/−^ mice.^10^ A recent report of a low resolution EM structure of heterotetrameric human complex I without NRBF2 suggests that the complex has some flexibility, but it is not clear what influence NRBF2 might have on this dynamic behavior.^13^

Our results show that depending on the relative abundance of NRBF2 to complex I, these proteins can make 2 types of assemblies. When complex I is more abundant than NRBF2, the predominant assembly is one NRBF2 dimer linking 2 complex I heterotetramers. When NRBF2 is in excess, it can form an additional assembly in which one NRBF2 dimer is bound to a single complex I. This behavior of NRBF2 contrasts with yeast Atg38 in which the Atg38 homodimer appears to make an asymmetric contact with complex I, without bridging 2 complexes. Although the NRBF2 MIT domain interacts with complex I, the C-terminal domain of NRBF2 is necessary to dimerize complex I. One consequence of NRBF2 forming a dimeric complex may be to increase the affinity of the complex I for membranes.

The low-resolution EM structure of the yeast Vps15-Vps34 subcomplex (Fig. S5) shows that the 2 components formed a stable core, and this assembly may also exist in mammalian cells.^18^ The EM density of the cross-linked human heterotetrameric and heteropentameric complex I agrees in dimensions with the previously reported V-shaped human tetrameric complex envelope.^13^

The HDX-MS data for the yeast complex I suggests that the coiled-coil I region in Atg14 (residues 25 to 36) is the main binding site for Atg38. Since Atg14 is the unique component that distinguishes heterotetrameric complex I from complex II, the HDX-MS results are consistent with a specific interaction between Atg38 and yeast complex I. The coiled-coil I in Vps30 was also protected from HDX in the presence of Atg38. Given the dimeric nature of Atg38, it is plausible that one Atg38 molecule binds to Atg14 and another to Vps30 within the same complex I. However, further structural analysis is necessary to reveal the detailed molecular architectures of complex I assemblies. HDX suggests that several regions in complex I are exposed in the presence of Atg38. This suggests that the binding of Atg38 may cause a conformational change in complex I, which may give access to membranes or binding proteins. These dynamic changes may be related to the structural flexibility reported for the human complex I.^13^

Although our HDX-MS data suggest that Atg38 binds to complex I mainly via the MIT domain, the C-terminal region of Atg38 was also protected from HDX (Fig. 3A). It is not clear whether this protection arises from direct binding to complex I or from conformational changes in Atg38. Nevertheless, the C-terminal region is important for binding to complex I in vivo (Fig. 6F). Higher resolution structural information will be required to understand the roles of the Atg38 C-terminal region upon binding to complex I.

### Functions of the C-terminal region of Atg38 during nitrogen starvation

The structure of the C-terminal region from Atg38 clearly shows the role of this domain in forming a parallel homodimer. Unlike its human ortholog NRBF2, Atg38 does not seem to dimerize yeast complex I. This suggests that the Atg38 homodimer is making an inherently asymmetric interaction with complex I. The structure of the homodimeric C-terminal domain of Atg38 shows striking asymmetry. It may be that this asymmetry only arises in the crystalline state of the domain; however, all 3 copies in the asymmetric unit are equivalent asymmetric dimers. Biologically relevant asymmetric dimers are rare, but one function that has been demonstrated for them is adapting a dimer for interaction with a single binding site.^19^ It may be that the asymmetric Atg38 C-terminal dimer makes an additional interaction with the asymmetric complex I.

In cells, it appears that Atg38 is responsible for incorporating complex I into larger cellular networks on membranes, especially under starvation conditions, as indicated by the ability of Atg38 to mediate complex I association with the P15 pellet in cell fractionation. A previous study suggests that the C-terminal region has additional, unidentified roles in autophagy beyond its role in homodimerization.^8^ We observed that deleting multiple segments simultaneously in the C-terminal region caused loss of dimerization and delocalization of the mutants from the PAS. However, even single segment deletions in the C-terminal region that remain dimeric caused their displacement from the membrane fraction (Fig. 6B). Our results suggest that the C-terminal domain of Atg38 is involved in interactions with complex I, localization to the PAS and possibly other cellular structures. Understanding how Atg38 and NRBF2 facilitate interactions of complex I with cellular components will have to await structures of these higher-order complexes.

## Materials and methods

### Constructs

All plasmids are listed Table S1. The full-length *ATG38* fragment used in Figs. 5 and 6 was from −997 bp to +737 bp including an intron. It was amplified by PCR using *S. cerevisiae* genomic DNA as a template and primers yoO568 (aaaaggtaccATGTCCAGTATTTACTCTACCTTG) and yoO472 (ttttgtcgaCTAGTTTTTTTGCTTATCCCTTCT). The *atg38* internal deletion fragments ((capΔ (123 to 181 deleted), stalkΔ (182 to 209 deleted), and capΔ+stalkΔ (123 to 209 deleted)) were generated from the full-length *ATG38*-carrying plasmid by PCR using appropriate primers. The fragments were ligated in the presence of T4 polynucleotide kinase (New England Biolabs, M0201), then further amplified with yoO568 and yoO472. The *atg38* C terminusΔ (residues 123 to 226 deleted), stalkΔ,+tailΔ (182 to 226 deleted), and tailΔ (209 to 226 deleted) fragments were directly obtained by PCR using the full-length *ATG38* as a template with appropriate primers. These PCR products were digested with KpnI+SalI, and inserted into pRS416-backbone vectors (pYO769 for 2xGFP encoded by a tandem repeat of EGFP (Clontech, 6085) or pYO484 for 3xFLAG). To generate plasmids expressing human complex I and complex I with *NRBF2* (pYO395 and pMO15, respectively), each subunit-coding fragment and EGFP coding fragment were cloned first into pcDNA3.1 (Invitrogen, V79020) or pcDNA4/TO (Invitrogen, V102020) individually. The plasmid carrying *VPS15* (*PIK3R4*) in pcDNA4/TO was then digested with MluI enzyme, and used as a backbone vector. A DNA fragment carrying the CMV/TO promoter-*VPS34* (*PIK3C3*)-BGH polyA site was amplified by PCR using the plasmid carrying *VPS34* in pcDNA4/TO as a template and primers opo825 (ctagtcaataatcaatgtcaacgcgcctagagccccagctggttc) and opo827 (cgcgatgtacgggccagatatacg). The PCR product was inserted into the digested *VPS15* vector using an In-Fusion reaction (Clontech, 639649). The resulting plasmid was again digested with MluI, and used as a backbone vector for the next round of Infusion reaction. The MluI digestion-In-Fusion cycles were repeated until all coding fragments with the promoter and the polyA site were inserted into single vectors. The final arrangement of genes in the pYO395 plasmid expressing human heterotetrameric complex I is ZZ-*ATG14*-EGFP-*BECN1-VPS34-VPS15*, and for the pMO15 plasmid expressing human heteropentameric complexes it is ZZ-*ATG14-NRBF2*-EGFP-*BECN1-VPS34-VPS15* (EGFP is used to monitor the efficiency of transfection).

For pYO1025 and pYO1022, a *VPS15*-2xTEV-ZZ fragment or a ZZ-3xTEV-*BECN1* fragment were cloned into a pCAG vector using In-Fusion reactions. Those plasmids were digested with SalI (for pYO1025) or SalI and SpeI (for pYO1022), and used as backbone plasmids for the next rounds of cloning. The DNA fragments carrying the CMV/TO promoter-*VPS34*-BGH polyA site or a CMV/TO promoter- ZZ-3xTEV-*ATG14*-BGH polyA site were amplified by PCR from the vectors described above using yoO940 (acatttccccgaaaagtgccacctgtgacattgattattgactagttatt) and yoO941 (gaccccgtaattgattactattaataccatagagcccaccgcat). The PCR products were inserted into the backbone plasmids using In-Fusion reactions.

### Yeast strains and cell growth

Yeast strains are listed in Table S2. For cell biology, yeast cells carrying plasmids (all in pRS416 backbone) were grown at 30°C in -URA medium (0.67% yeast nitrogen base without amino acids, 0.5% casamino acids and 2% glucose, supplemented with 0.002% adenine sulfate, 0.002% tryptophan, 0.002% tyrosine and 0.002% leucine; modified from Suzuki et al.^5^). To induce nitrogen starvation, the cells were incubated in SD(-N) medium (0.17% yeast nitrogen base without amino acid, without ammonium sulfate and with 2% glucose).

### Protein purification

Protein purification procedures for yeast complex I, Vps15-Vps34 dimer, and Vps15-Vps34^D731N^ (KI) dimer were described previously.^7^ The Vps15 (WD40Δ)-Vps34^D731N^ (KI) was purified using the same protocol as for the Vps15-Vps34 dimer purification.

Heterotetrameric and heteropentameric human PtdIns3K complexes were purified from HEK293T cells by transient transfection with pYO395 or pMO15. As a transfection agent, polyethyleneimine (PEI) (branched 25,000 Da, Polysciences, 24765) was used at a PEI:DNA ratio of 2:1. Transfection efficiency was assessed by observing the EGFP signal resulting from an additional EGFP expression cassette on the plasmid. Cells were harvested after 48 h.

For purification of both complexes for EM studies, HEK293T pellets were resuspended in lysis buffer (50 mM HEPES, pH 8.0, 150 mM NaCl, 2 mM EDTA, 1 mM EGTA, 0.5% CHAPS [Calbiochem, 220201], 8% glycerol, 1 mM DTT, 1x HALT protease inhibitor [Pierce, 78440]). Cells were lysed by sonication on ice for 2 min, followed by centrifugation to remove cell debris. The cleared lysate was added to IgG Sepharose 6 Fast Flow beads (GE Healthcare, 17096902) and agitated by rotation at 4°C. For elution of bound complexes, the ZZ-tag was cleaved by addition of in-house expressed and purified His-TEV protease for 1 h. Fractions containing complex were pooled and concentrated to approximately 0.4 mg/mL The complex was then stabilized and further purified by GraFix ultracentrifugation.^12,20^ For this purpose, 250 µL protein sample was loaded onto a 3.6 mL 20 to 30% glycerol gradient containing 0.0 to 0.15% glutaraldehyde (25 mM HEPES, pH 8.0, 200 mM NaCl, 1 mM DTT and 20 or 30% [v/v] glycerol). Gradients were centrifuged at 273,000 g in a Beckman SW60Ti rotor (Beckman Coulter, Krefeld, Germany) for 16 h at 4°C. 250 µL fractions were collected from the bottom and analyzed by SDS-PAGE. To identify fractions containing the complex, gradients without crosslinker were analyzed in parallel and these are presented in Fig. 2D.

For purifying human complex I for reconstitution, 5 L of FreeStyle 293-F (ThermoFisher, R79007) cells were grown in a WAVE Bioreactor system (GE Healthcare, CB0020L10-01, Little Chalfont, UK) to a cell density of 1.5 × 10^6^ cells/mL. Cells were then transfected with 2.8 mg of pYO1025 and 2.1 mg of pYO1022 using PEI at a PEI: DNA ratio of 2:1. Cells were harvested after 53 h, pelleted (15 min, 3000 g), frozen in liquid nitrogen, and stored at −80°C. To prepare protein, the cells were suspended in 150 mL of lysis buffer (50 mM Tris, pH 8.0, 200 mM NaCl, 1% Triton X-100 [Sigma, X100], 10% glycerol, 0.5 mM tris[2-carboxyethyl)phosphine (TCEP, Soltec Ventures, M115), 2 mM MgCl2, 1x EDTA-free inhibitor tablet (Roche, 05056489001)), and incubated on ice for 30 min. The Insoluble fraction was removed by centrifugation at 18,000 g for 30 min in an SS34 rotor (Thermo Fisher Scientific, Waltham, Massachusetts, USA). The supernatant fraction was incubated with 2 mL of IgG beads for 3.5 h. The beads with supernatant mixture were centrifuged at 1,000 g, and 100 ml of supernatant was removed. The IgG beads were transferred to a gravity flow column, and washed with 150 mL of wash buffer (50 mM Tris, pH 8.0, 200 mM NaCl, 0.1 % Triton X-100, 0.5 mM TCEP, 5 mM ATP [Sigma, A2383 ], 50 mM MgCl_2_, 5 µg/mL RNaseA [Sigma, 83834]) and 150 mL of TEV buffer (50 mM Tris, pH 8.0, 200 mM NaCl, 0.5 mM TCEP). A 10 mL aliquot of TEV buffer and 80 µL of 4.4 mg/mL TEV protease were added, and incubated at 4°C overnight without rotation. After the first TEV elution, 5 mL of TEV buffer was added and eluted again. In total, there was 10 mL overnight elution and 3 times of 5 mL post-overnight elution. The elution fractions were combined and concentrated using a 100 k concentrator (Millipore, UFC910096) for gel filtration. Gel filtration was done on an S200 10/30 column equilibrated with 20 mM Tris, pH 8.0, 200 mM NaCl, 0.5 mM TCEP. The main peak fractions were pooled and concentrated to 3.2 mg/mL. The proteins were frozen in liquid nitrogen and stored at −80°C. The gel filtration profile is consistent with an excess of BECN1 relative to other subunits of complex I.

Human NRBF2 was purified either as a GST-3C-NRBF2 fusion (pETM-33 backbone, EMBL Protein Expression and Purification Facility) or as a His6-NRBF2 fusion (pOPTH backbone, in-house). For purification of GST-3C-NRBF2, cell pellets were resuspended in lysis buffer (25 mM Tris, pH 7.5, 150 mM NaCl, 2 mM EDTA, 1 mM DTT, 1x EDTA-free protease inhibitor [Roche, 05056489001]) and lysed by 2 passages through a homogenizer (Avestin EmulsiFlex C3, Avestin, Mannheim, Germany). Cell debris was pelleted and the lysate was incubated with equilibrated glutathione Sepharose 4 Fast Flow resin (GE Healthcare, 17075605) for 2 h at 4°C under agitation. Fractions containing NRBF2 were pooled and 3C cleavage was performed overnight at 4°C. The cleaved protein was concentrated to 2 mg/mL with a 10-kDa cut-off spin concentrator (Millipore, UFC201024) and further purified by gel filtration on a Superdex 200 10/300 GL column (GE Healthcare, 17517501) in gel filtration buffer (50 mM Tris, pH7.5, 150 mM NaCl, 1 mM DTT). For the purification of the His6-NRBF2 full-length and His6-NRBF2-MIT domain (residues 1 to 89), cells were lysed in 25 mM Tris-HCl, pH 7.5, 0.3 M NaCl, 2 mM β-mercaptoethanol, 0.05 μg/mL Universal nuclease (Pierce, 88702), 0.5 mg/mL lysozyme (Sigma, L6876) and 10 mM imidazole (Sigma, 56749) by sonication for 10 min. After ultracentrifugation, supernatant fractions were filtered and loaded on a His-Trap FF 5 mL column (GE Healthcare, 17-5255-01) equilibrated in buffer A (20 mM Tris-HCl, pH 7.5, 0.3 M NaCl, 1 mM TCEP, 10 mM imidazole), washed with the same buffer and eluted with a gradient of 10 to 300 mM imidazole in 20 mM Tris-HCl, pH 7.5, 0.15 M NaCl, 1 mM TCEP. The peak fractions were pooled, concentrated and gel filtered on a Superdex 75 16/60 column equilibrated in 20 mM Tris-HCl, pH 7.5, 0.1 M NaCl, 0.5 mM TCEP. Peak fractions were pooled, concentrated, frozen in liquid N_2_ and stored at −80°C.

### Protein purification of Atg38

*S. cerevisiae* Atg38 constructs (full-length, N-terminal MIT domain (residues 1 to 78), the C-terminal fragment (residues 87 to 226) and various C-terminal truncation variants (C terminusΔ = residues 123 to 226 deleted, capΔ = 123 to 181 deleted, stalkΔ = 182 to 209 deleted, capΔ + stalkΔ = 123 to 209 deleted, stalkΔ+tailΔ = 182 to 226 deleted, tailΔ = 210 to 226 deleted) were expressed in *E. coli*. Cells were grown at 37°C to OD600 = 1.0, induced with 0.3 mM IPTG (Formedium, IPTG100) and incubated for a further 4 to 4.5 h at 37°C. Cells were resuspended in lysis buffer (25 mM Tris-HCl, pH 7.5, 0.2 M NaCl, 1 mM TCEP, 0.05 μg/mL Universal nuclease [Pierce, 88702), 0.5 mg/mL lysozyme and 1 mM PEFA [Melford, MB2003)) and sonicated for 6 min. After ultracentrifugation at 142,000 g for 45 min in a Ti45 rotor (Beckman Coulter, Krefeld, Germany), supernatant fractions were filtered and loaded on Glutathione Sepharose 4B resin (GE Healthcare, 17075605) equilibrated in 20 mM Tris-HCl, pH 7.5, 0.2 M NaCl, 2 mM DTT, washed extensively with the same buffer and either eluted with 10 mM glutathione, 50 mM Tris-HCl, pH 8.5, or cut with TEV protease, as needed. The full-length and N-terminal fragment of Atg38 were further purified on HiTrap Q column equilibrated in 20 mM Tris-HCl, pH 8.8, 1 mM TCEP and eluted with a NaCl gradient in the same buffer. After SDS-PAGE analysis, the relevant fractions were pooled and concentrated in Amicon Ultra 10-kDa cut-off concentrator (Millipore, UFC901024). All constructs were run on a S75 16/60 column equilibrated in 20 mM Tris-HCl, pH 8.8, 150 mM NaCl, 1 mM TCEP.

### Western blotting

For the preparation of total cell lysates in Fig. 6A, about 10 OD_600_ units of cells at midlog phase (OD_600_ = 0.8 to 1.0) were harvested in 1.5 mL tubes, flash frozen in liquid nitrogen, and stored at −80°C. The cells were treated using a cell wall loosening procedure (2 M LiAc on ice for 3 min followed by 0.4 M NaOH on ice for 3 min),^21^ and disrupted in 50 mM Tris (pH 8.0), 1% SDS, 8 M urea (VWR, 28877), 2 mM DTT, 5 mM EDTA with glass beads at 3,000 rpm for 30 sec with PowerLyzer homogenizer (MO BIO Laboratories, 13155, Carlsbad, California, USA) at 4°C. One volume of 4X sample buffer was added to 3 volumes of the lysates and loaded onto the gel. Proteins were detected using a mouse anti-Pgk1 antibody (Invitrogen, 459250) with anti-mouse-IgG HRP (Sigma, A9917), or an anti-Flag M2-HRP (Sigma, A8592). For detecting the His-tagged human NRBF2 MIT domain, Penta-His HRP conjugate Kit (Qiagen, 34660) was used with BenchMark (Invitrogen, 10747012) as a marker.

### Subcellular fractionation

Subcellular fractionation was performed using a modified version of a described procedure.^22^ Approximately 50 OD_600_ units of cells at midlog phase both in a standard growing condition and in nitrogen starvation condition were harvested, flash frozen in 2 mL tubes in liquid nitrogen, and stored at −80°C. Cells were suspended in 200 μL of lysis buffer (50 mM Tris, pH 8.0, 50 mM NaCl, 1 mM DTT, 0.5 mM PMSF, EDTA-free protease inhibitor tablet [Roche, 05056489001)) and glass beads. Cells were disrupted with a FastPrep-24 homogenizer (MP Biomedicals, 116004500, Santa Ana, California, USA) at 6.5 intensity for 45 sec. Cell debris, nucleus, and glass beads were removed by centrifuging at 1,000 g for 1 min. Supernatant fractions were transferred to new 1.5 mL tubes, and again centrifuged at 1,000 g for 5 min. The supernatant fractions from this spin represent the total fractions (T), and 150 μL of total was transferred to fresh 1.5 mL tubes and further spun at 15,000 g for 10 min to obtain S15 (supernatant) and P15 (pellet) fractions. P15 pellets were resuspended in 1% SDS, 8 M urea, 100 mM DTT.

### Immunoprecipitation

Cells were grown in 100 mL of -URA medium to midlog phase, then shifted to SD(-N) for 4 h at 30°C, harvested in 2 mL tubes, flash-frozen in liquid nitrogen, and stored at −80°C. Cells were suspended in lysis buffer (50 mM Tris, pH 8.0, 150 mM NaCl for Atg14-ZZ or 200 mM NaCl Atg38-ZZ, 2 mM β-mercaptoethanol, 12.5% glycerol, 1% Triton X-100, 0.5 mM PMSF, EDTA-free inhibitor tablet [Roche, 05056489001]) and disrupted with glass beads using a FastPrep-24 at 6.5 intensity for 45 sec. Cell debris and glass beads were removed by spinning at 20,000 g for 1 min, supernatant fractions were transferred to new 1.5 mL tubes, and centrifuged at 20,000 g for 2 min. Supernatant fractions were transferred to new 1.5 mL tubes, and 15 µL of anti-Flag M2 agarose beads (Sigma, A2220) were added and rotated at 4°C for 2 h. The beads were washed 5 times with 1 mL of lysis buffer, and resupended in SDS sample buffer. Both lysates and IP fractions were loaded on gels without boiling. Proteins were detected with an anti-Flag M2-HRP (Sigma, A8592), or a peroxidase anti-peroxidase complex (PAP, Sigma, P1291) antibody.

### HDX measurements of deuterium incorporation

Protein samples (10 μL of 2.5 μM protein in 20 mM Tris, pH 8.0, 50 mM NaCl, 2 mM TCEP) were mixed rapidly with 35 μL of D_2_O Buffer (96.6% D_2_O, 20 mM Tris, pH 8.0, 50 mM NaCl, 2 mM TCEP), producing a sample with a final concentration of 75% D_2_O. Four time points of exchange (3 sec, 30 sec, 300 sec and 3000 sec, at 23°C) were conducted, and the reactions were quenched with 20 μL of Quench Buffer (8.4% formic acid, 5 M guanidine-HCl), and immediately frozen in liquid nitrogen. Samples were stored at −80°C until mass spectrometry analysis.

Rapidly thawed samples were immediately injected onto an ultra performance liquid chromatography (UPLC) system described below immersed in ice. In order to digest the deuterated protein, the sample was passed through 2 immobilized pepsin Poroszyme columns (Applied Biosystems, 2-3131-00) at 150 μL/min for 3 min and the peptides were collected over a VanGuard C18 pre-column trap (Waters, 186003975). Peptic peptides were eluted from the trap by switching the flow to an in-line C18 UPLC column (Acquity 1.7 μm particle, 100 mm x 1 mm, Waters, 186002346). Peptic peptides were eluted from the column using a 20 min 5 to 45% gradient of buffer A (0.1% formic acid) and buffer B (100% acetonitrile), followed by a 4 min wash with 76% buffer B. Peptides eluted from the column were injected into a Xevo QTOF (Waters Corporation, Milford, MA, USA), acquiring for 30 min over an m/z range of 350 to 1500 using an electrospray ionization (ESI) source operated at a capillary temperature of 225°C, and a spray voltage of 3.5 kV.

### Peptide identification

Peptide identification was conducted by running tandem MS/MS experiments with a nondeuterated sample digested as above but with an extended, 60-min gradient over the UPLC column, rather than 20 min. An MS/MS was then conducted with a 20-min gradient, in order to correct the retention times for the identification runs done with the longer gradient. The MS tolerance was set to 15 ppm with an MS/MS tolerance of 0.2 Da. The resulting MS/MS datasets were analyzed using the Mascot search program within the Proteome Discoverer software package (Thermo Scientific). Peptides with a Mascot score less than 10 were excluded from further analysis. The remaining peptides were examined with HD-Examiner software (Sierra Analytics). The centroid value for each nondeuterated peptide was manually confirmed by searching the protein sample's MS scan to check for the correct m/z state, the presence of any overlapping peptides, and the correct retention time (any peptides failing these criteria were excluded from further analysis at the point).

### Mass analysis of peptide centroids

Validated nondeuterated peptides were subsequently analyzed for their deuterium incorporation (Figs. S6 to S16). Some overlapping peptides appeared due to a shift in m/z seen upon incorporation of deuterium and they were discarded from the data set. All peptides were once more validated for correct m/z and retention time. The deuterium levels referred to in the figures and the text are the relative levels of deuterium, as no fully deuterated sample was obtained. A mathematical correction was applied to compensate for the differences in the level of the deuterium in the exchange buffer (75%). The average error between replicate peptides was ≤0.5 Da. For our analysis, we considered as significant changes that were greater than 0.9 Da for both observations.

### Measurement of intact mass of Atg38 C terminusΔ

A 65 μL sample of 1.5 mg/ml of Atg38 C terminusΔ was precipitated using methanol and chloroform, sedimented, and resuspened in 100 μL of a 50% methanol and 1 % formic acid solution. This protein solution was then injected at a rate of 10 μL/min onto a Micromass LCT TOF (Waters Corporation, Milford, MA, USA), fitted with an ESI source, run with a sample cone voltage of 40 V, a capillary voltage of 2800 V, and a desolvation temperature of 80°C. The intact, denatured mass of the protein was determined using Micromass Maximum Entropy (MaxEnt) deconvolution software.

### Size exclusion chromatography coupled to multiangle light scattering (SEC-MALS)

The molecular masses in solution of yeast complex I, yeast complex I+Atg38, Atg38, human complex I, human complex I+NRBF2, NRBF2, and NRBF2 MIT domain were determined by SEC-MALS measurements using a Wyatt Heleos II 18 angle light scattering instrument coupled with a Wyatt Optilab rEX online refractive index detector (Wyatt, Goleta, California, USA). The molecular mass of NRBF2 in Fig. 2B was analyzed using a modular triple detector array (Viscotek TDA 305, Malvern Instruments Ltd., Malvern, UK) for online right-angle light scattering (RALS), refractive index and UV-vis determination calibrated using bovine serum albumin narrow standard. The protein samples were resolved on a S200 10/30, S75 10/30 (GE Healthcare, 17-1047-01), Superose 6 10/30 (GE Healthcare, 17-0537-01) or Superdex 200 10/300 increase (for NRBF2 in Fig. 2B) analytical gel filtration column (GE Healthcare, 28990944) running at 0.5 mL/min. The gel filtration buffers were 20 mM Tris, pH 8.8, 150 mM NaCl, 1 mM TCEP for complex I and complex I+Atg38, and 20 mM Tris, pH 7.9, 300 mM NaCl, 1 mM TCEP for Atg38, 20 mM Tris, pH 8.0, 200 mM NaCl, 0.5 mM TCEP for human complex I, human complex I+NRBF2, human complex I+NRBF2 MIT domain, NRBF2, NRBF2 MIT domain, and 50 mM Tris, pH 7.5, 150 mM NaCl, 1 mM DTT for NRBF2 in Fig. 2B. The protein concentrations were determined from the excess differential refractive index based on 0.186 RI increment for 1 g/mL protein solution. The concentration and the observed scattered intensity at each point in the chromatograms were used to calculate the absolute molecular mass using ASTRA 6 software (Wyatt) or Omnisec (Malvern) software.

### GST affinity isolation assays

The purified yeast complex I (at a final concentration of 1.78 μM) was mixed with 10-fold excess of GST, GST-Atg38 (1 to 226), GST-Atg38 (1 to 78), or GST-Atg38 (87 to 226). Initially, GST or GST fusion proteins were incubated on ice for 30 min with 20 µL of gluthathione Sepharose 4 Fast Flow resin, which had been equilibrated with reaction buffer (50 mM Tris, pH 8.8, 150 mM NaCl, 1 mM TCEP). The beads were washed once with 1 mL of reaction buffer, then the volume was adjusted to 90 µL. A 10 µL aliquot of 17.8 µM complex I was added to the beads, which were then incubated on ice for 90 min. The reactions were transferred to Poly-Prep Chromatography Columns (Bio-Rad, 7311550), and washed once with 10 mL reaction buffer by gravity flow. The columns were plugged, and 90 µL of reaction buffer was added to the resin to represent the bound fractions. For analysis, samples consisting of either 1 1:10 dilution of the input or the bound fractions were loaded on a SDS-Page gel. After electrophoresis, the gel was stained with InstantBlue (Expedeon, ISB1L).

### Reconstitution of human and yeast complex I with NRBF2/Atg38

Purified human complex I and purified human NRBF2 or NRBF2 MIT domain were mixed in gel filtration buffer (20 mM Tris, pH 8.0, 200 mM NaCl, 0.5 mM TCEP), incubated on ice for 30 min and loaded on a Superose 6 10/30 column. For 6:1 molar ratio of complex I to NRBF2 (full-length or MIT domain), 8.8 µM complex I was mixed with 1.47 µM NRBF2 in a 110 µL reaction and for 1:6 molar ratio of complex I to NRBF2, 8.8 µM complex I was mixed with 52.8 µM NRBF2 in a 110-µL reaction. For yeast complex reconstitution, yeast complex I and Atg38 were mixed in gel filtration buffer (20 mM Tris, pH 8.8, 150 mM NaCl, 0.5 mM TCEP), incubated on ice for 30 min and loaded on an S200 10/30 column. For 6:1 molar ratio of complex I to Atg38, 8.6 µM complex I was mixed with 1.4 µM Atg38 in a 120 µL reaction and for 1:6 molar ratio of complex I to Atg38, 8.6 µM complex I was mixed with 51.7 µM Atg38 in a 120 µL reaction. Fractions from gel-filtration runs were analyzed by SDS-PAGE and gels were stained with InstantBlue.

### Electron microscopy and image processing

A 4-µL aliquot of diluted full-length yeast Vps15-Vps34^D731N^ (KI) or Vps15 (WD40Δ) (1 to 981)-Vps34^D731N^ (KI) was applied to glow-discharged grids (Plano 300 mesh or Quantifoil R2/2) coated with a continuous carbon layer. After adsorption for 30 to 120 sec, the sample was stained by the droplet method using 2% (w/v) uranyl acetate.

For initial model reconstruction of the binary ScVps15 (WD40Δ)-Vps34 complex, 27 micrograph tilt-pairs of the Vps15Δ WD40-Vps34 complex were recorded at 0° and 55° stage tilt with a US4000 Gatan CCD camera (Pleasanton, California, USA) using an FEI Titan KRIOS (Hillsboro, Oregon, USA) operated at 120 kV. Micrographs were recorded at an underfocus between 1.0 and 1.5 µm at a magnification of 47,000 corresponding to a pixel size of 1.78 Å. In order to generate the initial structure of the truncated complex, we performed random-conical tilt (RCT) reconstruction.^23^ A total of 4,221 particle tilt pairs were windowed using e2RCTboxer^24,25^ and low-pass filtered to 20 Å. Using the untilted particle set, class averages were computed using EMAN2,^24,25^ each containing at least 60 members. Together with the tilted particle set, initial models were reconstructed for each class by EMAN2. After CTF correction by phase flipping,^26^ with additional untilted particles, a total of 6,891 particles were used for 3D structure refinement in EMAN2, using the RCT reconstructions low-pass filtered to 50 Å as initial models.

Images of the binary yeast Vps15-Vps34 complex were acquired on an FEI Polara microscope (Hillsboro, Oregon, USA) operated at 100 kV at a nominal underfocus of 1.0 µm and a magnification of 59,000 corresponding to a pixel size 1.91 Å recorded using a US4000 Gatan CCD camera. From a total of 670 micrographs (binned by a factor of 2), 14,172 particles were windowed semimanually in E2BOXER with a box size of 430 × 430 Å. The resulting particle stack was subjected to CTF estimation in EMAN2 and subsequently CTF corrected by phase flipping, followed by a band-pass filter operation from 20 Å to 200 Å. Reference-free 2D classification was performed by iterative multivariate statistical analysis (MSA) and alignment alternating between IMAGIC (Image Science Software GmbH, Berlin, Germany)^27^ and SPIDER software packages.^28^ Several cycles of classification and alignment were performed until convergence. After each iteration, the class averages were subjected to manual selection of nonredundant and detailed class averages, followed by alignment and masking, in order to prepare them as references for the following MSA iteration. Prior to refinement, the initial models from the RCT reconstruction and the refined truncated complex were low-pass filtered to 50 Å and 80 Å, respectively. Projection matching-based 3D structure refinement was performed with the entire particle set using SPIDER. Different initial models, obtained either from RCT reconstruction or the refined yeast Vps15 (WD40Δ)-Vps34 data set, converged to very similar refined 3D volumes of the Vps15-Vps34 complex. The resolution of the converging structure was determined at 28-Å resolution from the Fourier Shell Correlation (0.5 cutoff criterion) between 2 independent half-set reconstructions.

GraFix fractions of the human heterotetrameric and heteropentameric complexes were applied to carbon-coated grids and stained with 0.75% (w/v) uranyl formate as described above. Micrographs were acquired on an FEI Polara microscope operated at 100 kV at a magnification of 23,000 (5.1 Å/pixel) for the heterotetrameric complex and 31,000 (3.82 Å/pixel) for the heteropentameric complex with a US4000 CCD camera at an underfocus of 1 µm. A total of 372 micrographs were used to semiautomatically window 17,716 particles of the tetrameric complex, whereas for the pentameric complex 930 micrographs were acquired, of which 20,000 particles were semiautomatically boxed. Both particle sets were band-pass filtered from 25 to 400 Å and normalized, followed by iterative 2D classification as described above. Due to the observed structural heterogeneity and the limited particle set size, 3D structure refinement did not converge satisfactorily.

### Crystallization of Atg38

Crystals were obtained using the full-length Atg38 protein that was subjected to limited proteolysis with subtilisin and set up for crystallization without further purification: Atg38 (300 μL at 16 mg/mL in gel-filtration buffer containing 20 mM Tris-HCl, pH 8.8, 150 mM NaCl, 1 mM TCEP) was mixed with subtilisin (3 μL at 1 mg/mL; ProteAce kit [Hampton Research, HR2-429]), incubated for 10 h at 4°C and set up for crystallization without further purification. About 1800 crystallization conditions were screened using in-house nanoliter crystallization robotics,^29^ in 96-well MRC plates, by mixing 100 nL of protein with 100 nL of reservoir solution. The initial crystals were obtained from a Pi-minimal screen condition 3, containing 2 M ammonium nitrate, 0.150 M L-malate, pH 5.0 and 0.160 M magnesium sulfate (Jena Bioscience, CS-127).^30^ Optimal crystals were obtained from grids setup with a screen optimizer Dragonfly (TTP Labtech, Melbourn, UK), from a condition containing 1.97 M ammonium nitrate, 0.15 M (D/L) malate, pH 5.0 and 0.136 M magnesium sulfate. Ethylene glycol (2 µL of 30% stock) was added to the drop prior to crystal fishing. Selenomethionine-labeled crystals were obtained in similar conditions using standard protocols.

### Solving the Atg38 structure

Native and anomalous datasets were collected on beamline ID29 at the European Synchrotron Radiation Facility, diffracting respectively at 2.2 Å and 2.5 Å. Data sets were processed with XDS,^31^ and the phasing procedure was carried on peak and inflection point dataset using the PHENIX AutoSol program.^32^ The model obtained from experimental phases was refined against our best native data set at 2.2 Å using the PHENIX suite.

### Statistical analyses

All data were obtained from at least 3 independent experiments, and the results were presented as mean ± SD. Data were analyzed by one-way analysis of variance (ANOVA) followed by a multiple comparisons test (*P*<0.05), using Prism 7 for Mac OSX (GraphPad Software, Inc.).

### Accession numbers

The EM density of the yeast Vps15-34 complex and the corresponding PDB coordinates have been deposited to the Electron Microscopy Data Bank and Protein Data Bank with accession codes EMD-8235 and PDB-5KC2. The coordinates and diffraction data for the Atg38 C-terminal domain have been deposited to the Protein Data Bank, with accession code 5KC1.

## Supplementary Material

Supplementary files

## Abbreviations

ATG: autophagy related
BECN1: Beclin 1, autophagy related
EGFP: enhanced green fluorescent protein
EM: electron microscopy
GraFix: gradient fixation
GST: glutathione S-transferase
HDX-MS: hydrogen-deuterium exchange mass spectrometry
HEK293: human embryonic kidney293
KI: kinase inactive
MIT: microtubule-interacting and targeting
NRBF2: nuclear receptor-binding factor 2
PAS: phagophore assembly site
PCR: polymerase chain reaction
PEI: polyethyleneimine
PIK3C3: phosphatidylinositol 3-kinase catalytic subunit type 3, VPS34
PIK3R4: phosphoinositide 3-kinase regulatory subunit 4, VPS15
PtdIns3K: phosphatidylinositol 3-kinase
PtdIns3P: phosphatidylinositol 3-phosphate
SDS-PAGE: sodium dodecyl sulfate polyacrylamide gel electrophoresis
SEC-MALS: size exclusion chromatography coupled with multi-angle light scattering
TCEP: tris(2-carboxyethyl)phosphine
TEV: tobacco etch virus protease
UVRAG: UV radiation resistance-associated gene protein
Vps: vacuolar protein sorting
WD40: tryptophan aspartate containing repeat of about 40 residues
WT: wild type
