# Supplementary material for: Characterization of Atg38 and NRBF2, a fifth subunit of the autophagic Vps34/PIK3C3 complex
Source: Autophagy. 2016 Sep 14;12(11):2129–44. doi: 10.1080/15548627.2016.1226736 (PMC5103362; doi:10.1080/15548627.2016.1226736)
Supplement: Supplementary files [file kaup-12-11-1226736-s001.docx]

**Figure S1.** Reconstitution of yeast complex I with Atg38. Purified yeast complex I was mixed with purified Atg38 in the indicated molar ratios and analyzed on gel filtration on an S200 10/30 column (red and blue traces). For comparison, complex I alone (green) and Atg38 alone (black) were also run on the same column. The SDS PAGE analysis of the gel filtration fractions is shown below.

**Figure S2.** Reconstitution of human complex I with NRBF2. Purified human complex I was mixed with purified NRBF2 in the indicated molar ratios and run on gel filtration on a Superose 6 10/30 column (red and blue traces). SEC-MALS analysis of the peaks is shown above the traces with the average molecular mass indicated. The first peak in the chromatograms of complex I with NRBF2 (fractions 2 and 3, in the red and blue traces) has a size closest to two complex I assemblies linked by an NRBF2 homodimer (calculated mass 805 kDa). The apparent mass of material in this peak measured by MALS is 906 to 949 kDa for the blue trace and 835 to 949 kDa for the red trace. The very low levels (barely detectable by RI) of much larger species close to the void volume of the column eluting before this first peak contribute to the scattering intensity in this region, therefore resulting in slightly larger than expected masses. The second peak in the trace when the input has excess NRBF2 (fraction 5, red trace) has a size closest to one complex I bound to an NRBF2 homodimer (MALS measurement of the average mass is 481 kDa, with a range across the peak of 460 to 522 kDa. The expected mass for this assembly is 437 kDa). The MALS-estimated mass range for the NRBF2 dimer alone (green trace) is 67 to 68 kDa (calculated mass 69.6 kDa). SDS PAGE analysis of the gel filtration fractions is shown below. The purified complex I used for the reconstitution with NRBF2 appears to have an excess of ATG14-BECN1 that is present as a contaminant in the preparation.

**Figure S3.** Gel filtration analysis of the interaction of the NRBF2 MIT domain with human complex I. All samples were run on a Superose 6 10/30 column. When the input is complex I mixed with NRBF2 MIT domain at a molar ratio of 6:1 (magenta trace on the chromatogram, with the input visualized by SDS-PAGE, below, left), there is a single peak that elutes at a volume about the same as complex I alone (black trace). This peak has primarily complex I subunits. Some NRBF2 MIT domain is associated with the peak, as detected by western blotting (below). The green trace shows NRBF2 MIT domain run alone.

**Figure S4.** Representative negative-stain EM images for human complex I (**A**) and for human complex I with NRBF2 (**B**).

**Figure S5**. Negative-stain EM structure of yeast (Sc)Vps15-Vps34 subcomplex. (**A**) Electron micrograph of a D731N kinase-dead mutant Vps34 in a complex with Vps15. This mutant was more efficiently purified than the WT Vps15-Vps34, and was used for all of the EM work on this complex. The ScVps15-Vps34 heterodimers were visualized by negative stain. Characteristic particles are encircled. (**B**) Class averages revealed homogeneous particle preparations of this core subcomplex that possesses an elongated shape of 17 nm x 13 nm with a flexible protuberance of 7 nm, which introduces asymmetry to the subcomplex. Comparison of representative corresponding 2D class averages of the subcomplex containing full-length Vps15 (top) and a Vps15 deletion variant lacking the WD40 domain (bottom) suggests that the protuberance is the WD40 domain. (**C**) Comparison of representative class averages of ScVps15-Vps34 (top row) with the corresponding reprojection of the refined 3D structure (bottom row) shows very good agreement. (**D**) Fourier shell correlation (FSC) indicating a resolution of 28 Å according to the 0.5 criterion. (**E**) The 3D model of ScVps15-Vps34 has an elongated shape forming a slightly concave surface and reprojections are compatible with class averages. Four views of the ScVps15-Vps34 complex EM density (top) rotated around the long axis are shown. An atomic model for ScVps15-Vps34 was extracted from the X-ray crystal structure of yeast complex II (PDB entry 5DFZ) and is superimposed on the density (bottom). This model readily fits into the observed low-resolution EM density without any structural adjustments, suggesting that ScVps15-Vps34 forms a core scaffold for both complexes I and II.

**Figure S6.** A map of peptides analyzed by HDX-MS. Pepsin-digested peptide coverage map of yeast complex I. Identified and analyzed peptides are shown as bars below the primary sequence of individual proteins comprising complex I.

**Figure S7.** Global hydrogen-deuterium exchange for the Atg38 subunit alone. Column labels: S, first residue number for the peptide; E, last residue number for the peptide; Z, charge, #D, number of deuterons in the peptide; rt, retention time for the peptide; I, middle residue of the peptide; 3, 30, 300, 3000, seconds “on” exchange; Percent deuteration is indicated for each time point, with standard deviation (SD).

**% HDX of Atg38 in Pentamer**

**Figure S8.** Global hydrogen-deuterium exchange for Atg38 as a component of the Vps15-Vps34-Vps30-Atg14-Atg38 heteropentamer.

Columns as indicated in Figure S7.

**Figure S9.** Global hydrogen-deuterium exchange for Atg14 as a part of heterotetrameric complex I. Columns as indicated in Figure S7.

**Figure S10.** Global hydrogen-deuterium exchange for Atg14 as a part of the Vps15-Vps34-Vps30-Atg14-Atg38 heteropentamer. Columns as indicated in Figure S7.

**Figure S11.** Global hydrogen-deuterium exchange for Vps30 as a part of heterotetrameric complex I. Columns as indicated in Figure S7.

**Figure S12.** Global hydrogen-deuterium exchange for Vps30 as a part of the Vps15-Vps34-Vps30-Atg14-Atg38 heteropentamer. Columns as indicated in Figure S7.

**Figure S13.** Global hydrogen-deuterium exchange for Vps15 as a part of heterotetrameric complex I. Columns as indicated in Figure S7.

**Figure S14.** Global hydrogen-deuterium exchange for Vps15 as a part of the Vps15-Vps34-Vps30-Atg14-Atg38 heteropentamer. Columns as indicated in Figure S7.

**Figure S15.** Global hydrogen-deuterium exchange for Vps34 as a part of heterotetrameric complex I. Columns as indicated in Figure S7.

**Figure S16.** Global hydrogen-deuterium exchange for Vps34 as a part of the Vps15-Vps34-Vps30-Atg14-Atg38 heteropentamer. Columns as indicated in Figure S7.

**Figure S17.** Intact mass spectrum for the C terminus∆ construct of Atg38 (residues 123 to 226 deleted) purified from *E. coli*.

**Table S1.** Plasmids used in this study.

| ID | Construct | backbone | Use |
| --- | --- | --- | --- |
| pRS416 | empty | pRS416 | Cell biology |
| pYO780 | *ATG38* (FL)-2xEGFP |  |  |
| pYO816 | *atg38* (123 to 226Δ)-2xEGFP |  |  |
| pYO817 | *atg38* (182 to 226Δ)-2xEGFP |  |  |
| pYO835 | *atg38* (123 to 181Δ)-2xEGFP |  |  |
| pYO836 | *atg38* (182 to 209Δ)-2xEGFP |  |  |
| pYO842 | *atg38* (123 to 209Δ)-2xEGFP |  |  |
| pYO843 | *atg38* (210 to 226Δ)-2xEGFP |  |  |
| pYO785 | *ATG38* (FL)-3xFLAG |  |  |
| pYO814 | *atg38* (123 to 226Δ) -3xFLAG |  |  |
| pYO815 | *atg38* (182 to 226Δ)-3xFLAG |  |  |
| pYO827 | *atg38* (123 to 181Δ)-3xFLAG |  |  |
| pYO829 | *atg38* (182 to 209Δ)-3xFLAG |  |  |
| pYO844 | *atg38* (123 to 209Δ)-3xFLAG |  |  |
| pYO845 | *atg38* (210 to 226Δ)-3xFLAG |  |  |
| pYO225 | *ScVPS15*-x3 TEV-ZZ-*ADH1*t | pRS426 | Vps15-Vps34 dimer purification for EM, transformed with pYO290 |
| pYO286 | *Scvps15* (1-981)-x3 TEV-ZZ-*ADH1*t | pRS426 | Vps15 (WD40Δ)-Vps34 dimer purification for EM, transformed with pYO290 |
| pYO290 | *Scvps34* D731N | pRS424 | Vps15-Vps34 dimer purification for EM, transformed with pYO225 or pYO286 |
| pYO379 | *ScVPS34*+*ScVPS15*-3xTEV-ZZ-*ADH1*t+ZZ-3xTEV-*ScVPS30* | pRS426 | Complex I purification, transformed with pYO411 |
| pYO411 | ZZ-3xTEV-*ScATG14* | pRS424 | Complex I purification, transformed with pYO379 |
| pYO395 | ZZ-3xTEV-*HsATG14*+EGFP+*HsBECN1* +*HsPIK3C3*+*HsPIK3R4* | pcDNA4/TO | Human complex I coexpression for EM |
| pMO15 | ZZ-3xTEV-*HsATG14*+*HsNRBF2*+EGFP+*HsBECN1*+*HsPIK3C3*+*HsPIK3R4* | pcDNA4/TO | Human complex I+NRBF2 coexpression for EM |
| pOP764 | GST-TEV-*ScATG38* (FL) | pOP | Crystallization, GST affinity isolation |
| pOP775 | GST-TEV-*Scatg38* (1-78) | pOP | GST affinity isolation, MALS |
| pOP768 | GST-TEV-*Scatg38* (87-226) | pOP | GST affinity isolation |
| pOP795 | His6-TEV-*NRBF2* (FL, 1-287) | pOP | *In vitro* reconstitution, MALS |
| pOP796 | His6-TEV-*NRBF2* MIT domain (1-79) | pOP | *In vitro* reconstitution, MALS |
| pYO1089 | GST-TEV-*Scatg38*, (123 to 226Δ) | pOP | MALS |
| pYO1090 | GST-TEV-*Scatg38* (182 to 226Δ) | pOP | MALS |
| pYO1091 | GST-TEV-*Scatg38* (123 to 181Δ) | pOP | MALS |
| pYO1092 | GST-TEV-*Scatg38* (182 to 209Δ) | pOP | MALS |
| pYO1093 | GST-TEV-*Scatg38* (123 to 209Δ) | pOP | MALS |
| pYO1094 | GST-TEV-*Scatg38* (210 to 226Δ) | pOP | MALS |
| pYO1025 | *HsPIK3C3*+*HsPIK3R4*-2xTEV-ZZ | pCAG | Human complex I expression for *in vitro* reconstitution, cotransfected with pYO1022 |
| pYO1022 | ZZ-3xTEV-*HsATG14*+ZZ-3xTEV-*HsBECN1* | pCAG | Human complex I expression for *in vitro* reconstitution, cotransfected with pYO1025 |
| C134 | GST-3C-*HsNRBF2* | pETM33 | MALS, *in vitro* reconstitution |

All yeast protein expression plasmids are under the *GAL1* promoter (a kind gift from Kiyoshi Nagai, MRC Laboratory of Molecular Biology) and the *MFA1* terminator, except where noted as *ADH1*t, in which case the *ADH1* terminator was used. All cell biology plasmids except pRS416 are under the *ATG38* promoter from -997 bp to -1 bp and the *ADH1* terminator. All mammalian subunits were under the *CMV/TO* or *CAG* promoter and the *BGH* (bovine growth hormone) or rabbit beta-globin polyA site. EGFP was under the *CMV* promoter and the *BGH* polyA site.

**Table S2.** Yeast strains used in this study.

| ID | Genotype | Reference, source |
| --- | --- | --- |
| BY4741 | *MATa his3Δ1 leu2Δ0 met15Δ0 ura3Δ0* | Open Biosystem |
| *atg38∆* | BY4741 *atg38Δ::KanMX* | Open Biosystems |
| JWY104 | *MATa, pra1-1, prb1-1, prc1-1, cps1-3, ura3-Δ5, leu2-3,-112, his-* | 1 |
| Bcy123 | *pep4::HIS3 prb::LEU2 bar1:HISG lys2::GAL1/10-GAL4 can1 ade2 ura3 leu2–3,112 trp1* | 2 |
| YOY204 | JWY104 *ATG38-ZZ::KanMX* | This study |
| YOY361 | Bcy123 *ATG14-ZZ::KanMX atg38∆::NatMX* | This study |

**References**

1.	Whyte JR, Munro S. The Sec34/35 Golgi transport complex is related to the exocyst, defining a family of complexes involved in multiple steps of membrane traffic. Dev Cell 2001; 1:527–37.

2.	Galej WP, Oubridge C, Newman AJ, Nagai K. Crystal structure of Prp8 reveals active site cavity of the spliceosome. Nature 2013; 493:638–43.
